# Race/Ethnicity may be an Important Predictor of Life Expectancy in Localized Prostate Cancer Patients: Novel Analyses Using Social Security Administration Life Tables

**DOI:** 10.1007/s40615-022-01257-y

**Published:** 2022-02-18

**Authors:** Christoph Würnschimmel, Luigi Nocera, Mike Wenzel, Claudia Collà Ruvolo, Zhe Tian, Fred Saad, Alberto Briganti, Shahrokh F. Shariat, Vincenzo Mirone, Felix K. H. Chun, Derya Tilki, Markus Graefen, Pierre I. Karakiewicz

**Affiliations:** 1 Martini-Klinik Prostate Cancer Center, University Hospital Hamburg-Eppendorf, Martinistraße 52, 20246 Hamburg, Germany; 2 Cancer Prognostics and Health Outcomes Unit, Division of Urology, University of Montréal Health Center, Montréal, Québec Canada; 3 Department of Urology, Lucerne Cantonal Hospital, Lucerne, Switzerland; 4 Department of Urology and Division of Experimental Oncology, URI, Urological Research Institute, IBCAS San Raffaele Scientific Institute, Milan, Italy; 5 Department of Urology, University Hospital Frankfurt, Frankfurt am Main, Germany; 6 Department of Neurosciences, Reproductive Sciences and Odontostomatology, University of Naples Federico II, Naples, Italy; 7 Department of Urology, Comprehensive Cancer Center, Medical University of Vienna, Vienna, Austria; 8 Departments of Urology, Weill Cornell Medical College, New York, NY USA; 9 Department of Urology, University of Texas Southwestern, Dallas, TX USA; 10 Department of Urology, Second Faculty of Medicine, Charles University, Prague, Czech Republic; 11 Institute for Urology and Reproductive Health, I.M. Sechenov First Moscow State Medical University, Moscow, Russia; 12 Division of Urology, Department of Special Surgery, Jordan University Hospital, The University of Jordan, Amman, Jordan

**Keywords:** Life expectancy prediction, Social Security Administration, Life table, Localized prostate cancer, SEER

## Abstract

**Purpose:**

To test the effect of race/ethnicity on Social Security Administration (SSA) life tables’ life expectancy (LE) predictions in localized prostate cancer (PCa) patients treated with either radical prostatectomy (RP) or external beam radiotherapy (EBRT). We hypothesized that LE will be affected by race/ethnicity.

**Patients and Methods:**

We relied on the 2004–2006 Surveillance, Epidemiology, and End Results database to identify D’Amico intermediate- and high-risk PCa patients treated with either RP or EBRT. SSA life tables were used to compute 10-year LE predictions and were compared to OS. Stratification was performed according to treatment type (RP/EBRT) and race/ethnicity (non-Hispanic White, non-Hispanic Black, Hispanic/Latino, and Asian).

**Results:**

Of 55,383 assessable patients, 40,490 were non-Hispanic White (RP 49.3% vs. EBRT 50.7%), 7194 non-Hispanic Black (RP 41.3% vs. EBRT 50.7%), 4716 Hispanic/Latino (RP 51.0% vs. EBRT 49.0%) and 2983 were Asian (RP 41.6% vs. EBRT 58.4%). In both RP and EBRT patients, OS exceeded life tables’ LE predictions, except for non-Hispanic Blacks. However, in RP patients, the magnitude of the difference was greater than in EBRT. Moreover, in RP patients, OS of non-Hispanic Blacks virtually perfectly followed predicted LE. Conversely, in EBRT patients, the OS of non-Hispanic Black patients was worse than predicted LE.

**Conclusions:**

When comparing SEER-derived observed OS with SSA life table–derived predicted life expectancy, we recorded a survival disadvantage in non-Hispanic Black RP and EBRT patients, which was not the case in the three other races/ethnicities (non-Hispanic Whites, Hispanic/Latinos, and Asians). This discrepancy should ideally be confirmed within different registries, countries, and tumor entities. Furthermore, the source of these discrepant survival outcomes should be investigated and addressed by health care politics.

## Background

Life expectancy (LE) needs to be taken into account in the localized prostate cancer (PCa) clinical decision-making process [1, 2], especially when curative management such as radical prostatectomy (RP) or external beam radiotherapy (EBRT) is considered. For example, the European Association of Urology (EAU) recommends active treatment for PCa only in patients with a LE above 10 years [1]. In this regard, the National Comprehensive Cancer Network (NCCN) guidelines endorse the use of age-based Social Security Administration (SSA) life tables for LE prediction in North American PCa patients [3, 4]. Although the SSA life tables were validated in general, the effect of race/ethnicity on LE has not been examined [5]. Nevertheless, evidence suggests that race/ethnicity may be a determinant of LE [6, 7] and this hypothesis has not been tested in the setting of localized PCa. To address this void, we tested for differences between observed overall survival (OS) and SSA life tables’ predicted LE according to four racial/ethnic groups: non-Hispanic Whites, non-Hispanic Blacks, Hispanic/Latinos, and Asians. Additionally, we stratified according to treatment type, since LE characteristics at RP and EBRT are also known to vary [8, 9]. We hypothesized that SSA life table–derived LE predictions differ from OS rates between the four racial/ethnic groups.

## Patients and Methods

We identified non-Hispanic White, non-Hispanic Black, Hispanic/Latino, and Asian patients with D’Amico intermediate- to high-risk localized PCa treated with either RP or EBRT between 2004 and 2006 and who have available follow-up of 10 years within the Surveillance, Epidemiology, and End Results (SEER) database. D’Amico intermediate-risk was defined as clinical T-stage 2b (cT2b) and/or prostate-specific antigen (PSA) of 10–20 ng/ml and/or Gleason Grade Group (GGG) 3. D’Amico high risk was defined as clinical T-stage ≥ cT2c, PSA > 20 ng/ml and/or GGG ≥ 4 [10]. We relied on intermediate/high-risk patients in order to focus the analyses on a patient cohort that would most likely resemble the optimal patient cohort for active treatment with RP or EBRT [11, 12]. Clinical node positive status (cN1) was allowed within analyses regardless of D’Amico risk grouping. Furthermore, adjuvant or salvage EBRT following RP, which included all procedures within 1 year after diagnosis, was allowed within all analyses. We excluded patients with unknown metastatic status (*n* = 2965), unknown race/ethnicity information (*n* = 1354), and Native American race/ethnicity (*n* = 354). These selection criteria resulted in a cohort of 55,383 assessable patients. Finally, the race/ethnicity information provided by SEER stems from 18 different cancer registries in the USA, which were all included in the present analysis and which needs to be considered in the light of potential discrepancies in accrual of data and lack of standardization when classifying race/ethnicity status [13–15].

### Statistical Analyses

Monte Carlo simulation was used to create a simulated cohort resembling the exact age composition of the actual 2004–2006 SEER database population of 55,383 men with localized PCa, according to previous methodology [5]. Based on SSA life tables’ predictions for a 10-year span up to the year 2016 (henceforth referred to as “predicted LE”), a Markov chain representing natural progression of age was constructed for each individual’s age. Within the Markov chain, each simulated patient could either survive or die within each of ten simulated year intervals. For each examined scenario, the model provided a 10-year LE probability. The latter was included in Kaplan–Meier plots and compared with OS rates according to treatment type (RP and EBRT) and according to race/ethnicity (non-Hispanic White, non-Hispanic Black, Hispanic/Latino, and Asian). Furthermore, the differences OS and predicted LE for each year were calculated and plotted.

R software environment for statistical computing and graphics (version 3.4.0 for MAC OS X; http://www.r-project.org/) was used for all statistical analyses [16]. Descriptive statistics included frequencies and proportions for categorical variables. Medians and interquartile-ranges (IQR) were reported for continuously coded variables. Chi-square and log-rank tested the statistical significance in proportions and survival differences. All tests were two-sided with a level of significance set at *p* < 0.05.

## Results

### Study Population

**Fig. 1:**
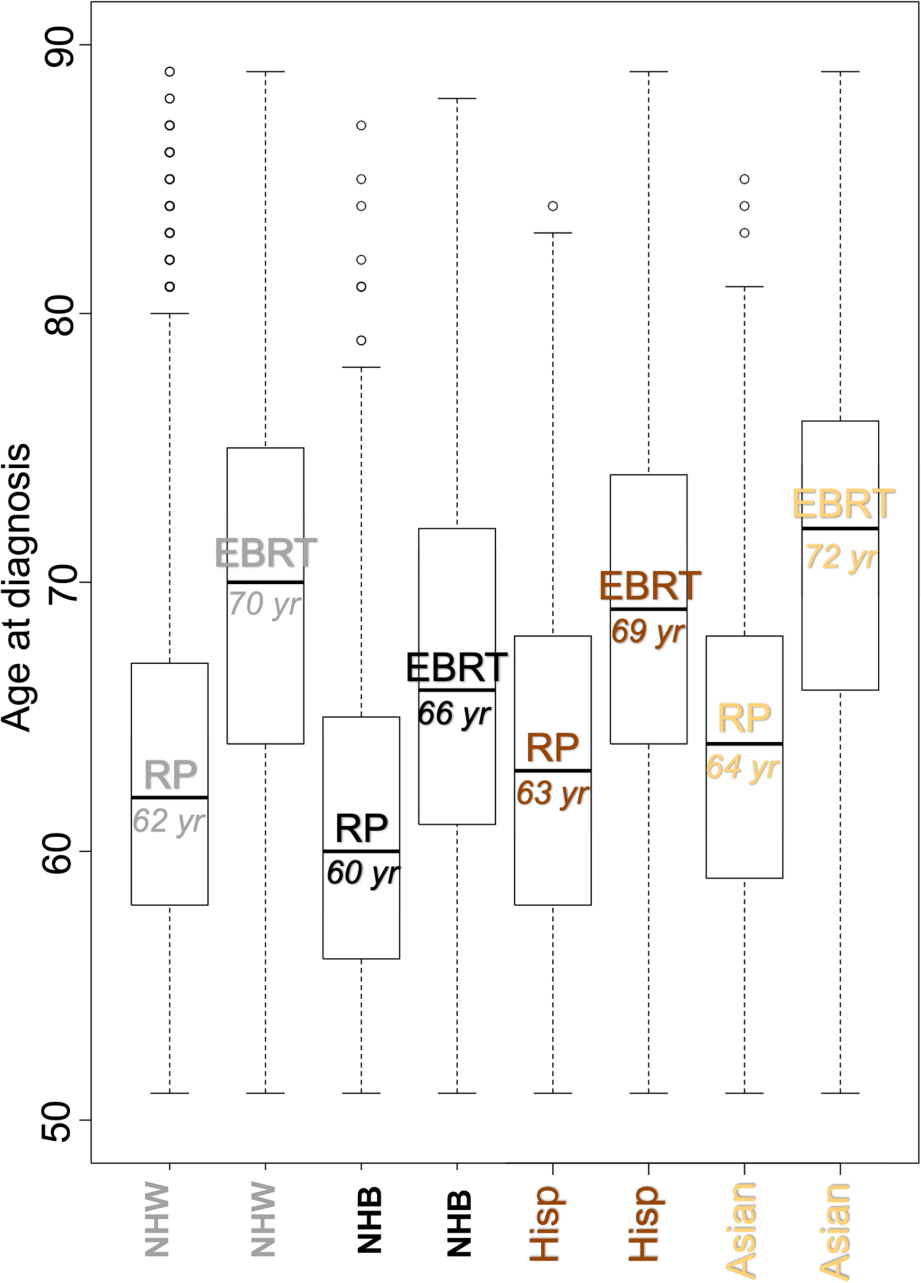
Box plots displaying age distribution between racial/ethnic groups (non-Hispanic White (NHW), non-Hispanic Black (NHB), Hispanic/Latino (Hisp), and Asian) treated with either radical prostatectomy (RP) or external beam radiotherapy (EBRT)

Of 55,383 eligible patients with intermediate- or high-risk PCa, 40,490 were non-Hispanic White, 7194 non-Hispanic Black, 4716 Hispanic/Latino, and 2983 were Asian (Table 1). RP patients were in general younger (62 years, interquartile range IQR 57–67) than EBRT patients (70 years, IQR 64–75). Median ages at diagnosis for RP/EBRT were 62 years/70 years, 60 years/66 years, 63 years/69 years, and 64 years/72 years for non-Hispanic Whites, non-Hispanic Blacks, Hispanic/Latinos, and Asians, in that order (Fig. 1).

**Table 1:** Descriptive characteristics of non-Hispanic White (NHW), non-Hispanic Black (NHB), Hispanic/Latino, and Asian patients diagnosed with localized prostate cancer in the Surveillance, Epidemiology, and End Results database 2004–2006. Abbreviations: *PSA*, prostate-specific antigen, *GGG* Gleason Grade Group, *RP* radical prostatectomy, *EBRT* external beam radiotherapy, *IQR* interquartile range

	Overall *n* = 55,383	NHW *n* = 40,490	NHB *n* = 7194	Hispanic/Latino *n* = 4716	Asian *n* = 2983
Age (year), median (IQR)	66 (60–72)	66 (60–72)	64 (58–69)	66 (60–71)	68 (63–74)
PSA (ng/ml), median (IQR)	6.9 (4.9–11.3)	6.6 (4.8–10.7)	7.9 (5.3–13.8)	7.8 (5.3–12.8)	8.3 (5.7–13.2)
Tumor stage, *n* (%)					
cT1	25,563 (46.2)	18,093 (44.7)	3836 (53.3)	2148 (45.5)	1486 (49.8)
cT2	27,576 (49.8)	20,739 (51.2)	3068 (42.6)	2399 (50.9)	1370 (45.9)
cT3	1968 (3.6)	1477 (3.6)	242 (3.4)	138 (2.9)	111 (3.7)
cT4	220 (0.4)	152 (0.4)	35 (0.5)	21 (0.4)	12 (0.4)
cTx	56 (0.1)	29 (0.1)	13 (0.2)	10 (0.2)	4 (0.1)
GGG, *n* (%)					
I	13,986 (25.3)	10,268 (25.4)	1732 (24.1)	1320 (28.0)	666 (22.3)
II	22,667 (40.9)	16,670 (41.2)	3013 (41.9)	1879 (39.8)	1105 (37.0)
III	8386 (15.1)	6096 (15.1)	1116 (15.5)	661 (14.0)	513 (17.2)
IV	5846 (10.6)	4189 (10.3)	804 (11.2)	476 (10.1)	377 (12.6)
V	3934 (7.1)	2863 (7.1)	434 (6.0)	335 (7.1)	302 (10.1)
Unknown	564 (1.0)	404 (1)	95 (1.3)	45 (1.0)	20 (0.7)
Lymph node status, *n* (%)					
cN0	53,804 (97.1)	39,347 (97.2)	6997 (97.3)	4561 (96.7)	2899 (97.2)
cN1	905 (1.6)	658 (1.6)	125 (1.7)	84 (1.8)	38 (1.3)
cNX	674 (1.2)	485 (1.2)	72 (1.0)	71 (1.5)	46 (1.5)
D’Amico risk group, *n* (%)					
Intermediate	35,494 (64.1)	26,252 (64.8)	4389 (61.0)	3048 (64.6)	1805 (60.5)
High	19,889 (35.9)	14,238 (35.2)	2805 (39.0)	1668 (35.4)	1178 (39.5)
Treatment type, *n* (%)					
RP	26,568 (48)	19,949 (49.3)	2973 (41.3)	2405 (51.0)	1241 (41.6)
EBRT	28,815 (52)	20,541 (50.7)	4221 (58.7)	2311 (49.0)	1742 (58.4)

At diagnosis, median PSA values (in ng/ml) for RP patients were 5.9, 6.6, 6.7, and 7.0 respectively for non-Hispanic Whites, non-Hispanic Blacks, Hispanic/Latinos, and Asians. For EBRT patients, PSA values were 7.4, 8.8, 8.9, and 9.5, respectively, for non-Hispanic Whites, non-Hispanic Blacks, Hispanic/Latinos, and Asians. Within all four racial/ethnic groups, both RP and EBRT patients predominantly harbored GGG 1–3 (81.3% overall) and cT1-2 stages (96.0% overall), regardless of treatment type (Table 1). The rates of adjuvant or salvage EBRT after RP were 4.1, 4.1, 4.4, and 4.7% and the rates of salvage RP after EBRT were 0.1, 0.1, 0.1, and 0.4% among non-Hispanic Whites, non-Hispanic Blacks, Hispanic/Latinos, and Asians, in that order.

### Observed Overall Survival Versus Predicted Life Expectancy in Radical Prostatectomy Patients

**Fig. 2:**
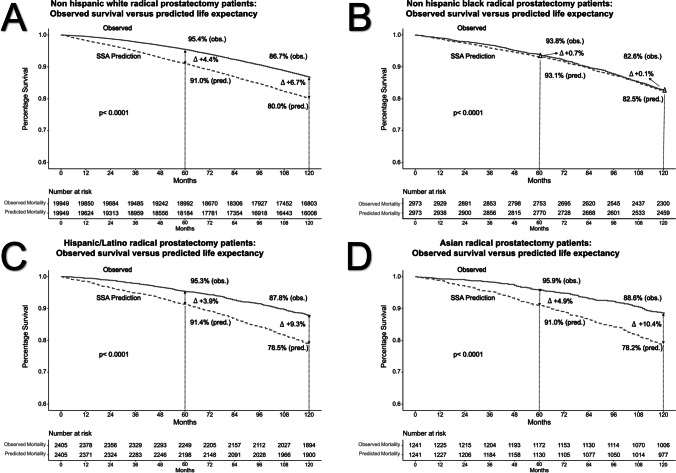
Kaplan–Meier curves of OS within non-Hispanic White (**A**), non-Hispanic Black (**B**), Hispanic/Latino (**C**), and Asian (**D**) localized prostate cancer patients in the Surveillance, Epidemiology, and End Results database (2004–2006) treated by radical prostatectomy compared to predicted life expectancy derived by the Social Security Administration (SSA) life tables

The comparison between OS and predicted LE at RP was stratified according to four racial/ethnic groups: non-Hispanic Whites (Fig. 2A), non-Hispanic Blacks (Fig. 2B), Hispanic/Latinos (Fig. 2C), and Asians (Fig. 2D). For example, in non-Hispanic Whites treated with RP, OS was 95.4% at 5 years and 86.7% at 10 years. For the same timepoints, the respective predicted LE values were 91.0% and 80.0%. OS versus predicted LE values resulted in a difference of + 4.4% at 5 years and + 6.7% at 10 years, favoring OS over predicted LE. The same analysis in non-Hispanic Blacks treated with RP yielded virtually the same OS and predicted LE values (OS at 5 years: 93.8%, 10 years: 82.6% versus predicted LE at 5 years: 93.1%, 10 years: 82.5%). These respectively resulted in virtually no differences at 5 years (+ 0.7%) and 10 years (+ 0.1%). In Hispanic/Latinos treated with RP, the OS was 95.3% at 5 years and 87.8% at 10 years. For the same timepoints, the respective predicted LE values were 91.4% and 78.5%. The OS versus predicted LE values resulted in a difference of + 3.9% at 5 years and + 9.3% at 10 years, favoring OS over predicted LE. In Asian patients treated with RP, the OS was 95.9% at 5 years and 88.6% at 10 years. For the same timepoints, the respective predicted LE values were 91.0% and 78.2%. The OS versus predicted LE values resulted in a difference in + 4.9% at 5 years and + 10.4% at 10 years, favoring OS over predicted LE.

**Fig. 3:**
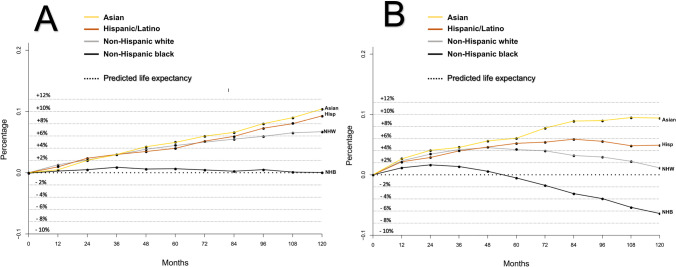
Differences between Social Security Administration (SSA) life tables’ predicted life expectancy (dotted line) and observed survival (solid lines) at different timepoints within a population of localized prostate cancer patients from non-Hispanic White (NHW), Hispanic/Latino, non-Hispanic Black (NHB), and Asian patients treated by radical prostatectomy (**A**) or external beam radiotherapy (**B**) within the Surveillance, Epidemiology, and End Results database (2004–2006)

Figure 3A combines the recorded differences between OS versus predicted LE within the four examined racial/ethnic groups treated with RP. For non-Hispanic Blacks, the plotted line illustrating the difference between OS and predicted LE closely corresponds to the horizontal line and indicates a negligible difference between these two values. Conversely, the plotted differences between OS and predicted LE for non-Hispanic Whites vs. Hispanic/Latinos vs. Asians indicate that OS invariably exceeded predicted LE. This phenomenon is evidenced by positive values denoting the difference between OS and predicted LE. Among these three racial/ethnic groups (non-Hispanic White, Hispanic/Latinos, Asians) the greatest difference between OS and predicted LE (favoring OS) at 10 years was recorded in Asians (+ 10.4%), followed by Hispanic/Latinos (+ 9.3%), and non-hispanic whites (+ 6.7%), in that order

### Observed Overall Survival Versus Predicted Life Expectancy in External Beam Radiotherapy Patients

**Fig. 4:**
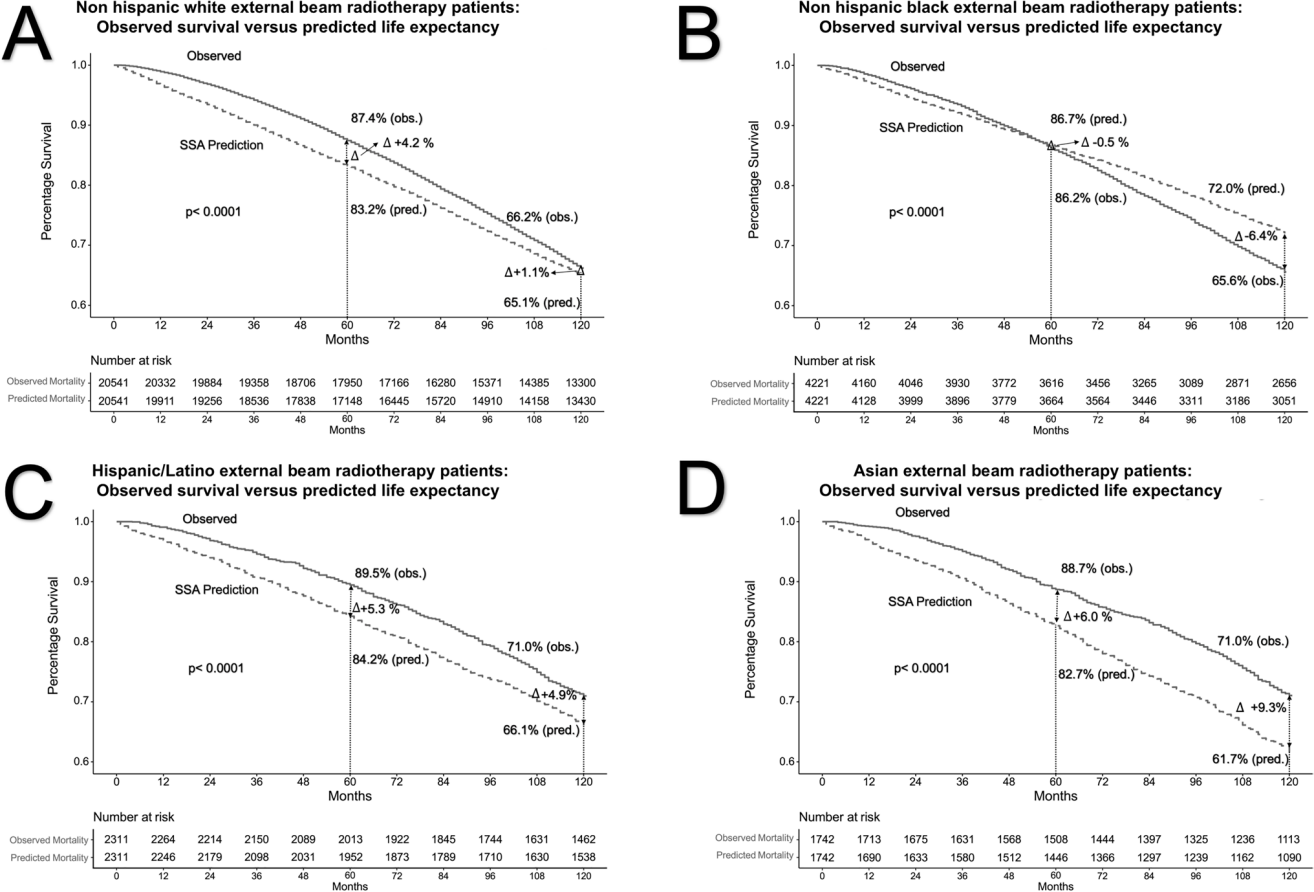
Kaplan–Meier curves of OS within non-Hispanic White (**A**), non-Hispanic Black (**B**), Hispanic/Latino (**C**), and Asian (**D**) localized prostate cancer patients in the Surveillance, Epidemiology, and End Results database (2004–2006) treated by external beam radiotherapy compared to predicted life expectancy derived by the Social Security Administration (SSA) life tables

The comparison between OS and predicted LE at EBRT was also stratified according to four racial/ethnic groups: non-Hispanic Whites (Fig. 4A), non-Hispanic Blacks (Fig. 4B), Hispanic/Latinos (Fig. 4C), and Asians (Fig. 4D). In non-Hispanic Whites treated with EBRT, the OS was 87.4% at 5 years and 66.2% at 10 years. For the same timepoints, the respective predicted LE values were 83.2% and 65.1%. The OS versus predicted LE values resulted in a difference of + 4.2% at 5 years and + 1.1% at 10 years, favoring OS over predicted LE. In non-Hispanic Blacks treated with EBRT, the OS was 86.2% at 5 years and 65.6% at 10 years. For the same timepoints, the respective predicted LE values were 86.7% and 72.0%. The OS versus predicted LE values resulted in a difference of − 0.5% at 5 years and − 6.4% at 10 years, favoring predicted LE over OS. In Hispanic/Latinos treated with EBRT, the OS was 89.5% at 5 years and 71.0% at 10 years. For the same timepoints, the respective predicted LE values were 84.2% and 66.1%. The OS versus predicted LE values resulted in a difference of + 5.3% at 5 years and + 4.9% at 10 years, favoring OS over predicted LE. In Asians treated with EBRT, the OS was 88.7% at five years and 71.0% at ten years. For the same timepoints, the respective predicted LE values were 82.7% and 61.7%. The OS versus predicted LE values resulted in a difference in + 6.0% at five years and + 9.3% at ten years, favoring OS over predicted LE.

Figure 3B combines the recorded differences between observed survival versus predicted LE within the four examined racial/ethnic groups treated with EBRT. For non-Hispanic Blacks, the plotted line illustrating the difference between OS and predicted LE exhibits a lower OS compared to their respective predicted LE from 5 years onwards. Conversely, the plotted differences between OS and predicted LE between non-Hispanic Whites, Hispanic/Latinos, and Asians invariably favored observed survival over predicted LE. This phenomenon is evidenced by positive values denoting the difference between OS and predicted LE. Among these three racial/ethnic groups (non-Hispanic White, Hispanic/Latinos, Asians), the greatest difference between OS and predicted LE (favoring OS) at 10 years was recorded in Asians (+ 9.3%), followed by Hispanic/Latinos (+ 4.9%) and non-Hispanic Whites (+ 1.1%), in that order

## Discussion

We hypothesized that SSA life table–derived LE predictions differ to OS rates between racial/ethnic groups [6, 7]. Our analysis revealed several noteworthy findings.

First, in RP patients, we invariably recorded better OS than that predicted by SSA life tables. The exception to this rule consisted of non-Hispanic Black patients, whose OS virtually perfectly corresponded to their respective LE prediction. It is of interest that Asian, Hispanic/Latino, and non-Hispanic white RP patients exhibited comparable patterns of OS that exceeded their respective LE predictions to a similar extent. In contrast, non-Hispanic Black RP patients’ OS exhibited virtually no departures from their predicted LE. This observation indicates that overall survival of non-Hispanic Blacks is worse than that of other racial/ethnic groups. This is applicable, even in the context of younger age of non-Hispanic Blacks relative to the three other racial/ethnic groups.

Second, the above observations indicate that RP patients, except for non-Hispanic Blacks, exhibit better OS than the general North American population, from which LE predictions are derived. An explanation for the discrepancy between OS of non-Hispanic Blacks versus other racial/ethnic groups can be proposed. It is possible that the general health of non-Hispanic Blacks as a group is worse than that of the three other racial/ethnic groups and represents the determinant of subsequent survival. This observation is worrisome and may be indicative of the need to correct for potential general health disadvantages in non-Hispanic Blacks, including those treated for localized PCa. Worse general health of non-Hispanic Blacks has been previously reported [17–20]. However, to the best of our knowledge, no previous publication contrasted SSA life table–derived predicted LE with OS. In consequence, no previous investigators were able to quantify the overall survival detriment relative to predicted LE in non-Hispanic Blacks. Furthermore, no other investigators contrasted the figures recorded in non-Hispanic Blacks with those recorded for other racial/ethnic groups.

Third, we also examined differences between OS and predicted LE in EBRT patients. Our findings were similar to those described for RP patients. Specifically, OS for Asian, Hispanic/Latino, and non-Hispanic White EBRT patients, in general, exceeded that of their predicted LE. However, relative to RP patients, the overall survival benefit was of smaller magnitude. In EBRT patients, the difference between OS and predicted LE in Hispanic/Latinos was roughly half of the benefit recorded in Asians and the long-term survival advantage of non-Hispanic Whites only corresponded to a fraction of that recorded in Asians. These observations are different from those recorded in RP patients where Asians, Hispanic/Latinos, and non-Hispanic Whites exhibited better OS than respective predicted LE to very similar extents. These differences possibly suggest that general health, which determines OS in these three racial/ethnic groups, differs more appreciably in EBRT patients than in RP patients. Nonetheless, all three racial/ethnic groups (Asians, Hispanic/Latinos, and non-Hispanic Whites) treated with EBRT invariably demonstrate better OS than predicted LE. This phenomenon was not applicable to non-Hispanic Black EBRT patients. Not only did they exhibit worst OS of all examined EBRT racial/ethnic groups (as was also observed in RP patients), but also exhibited worse OS than that of their respective predicted LE as of 5 years of follow-up.

In summary, the SSA life table–derived LE predictions underestimate the OS of Asian, Hispanic/Latino, and non-Hispanic White RP and EBRT patients. The degree of LE underestimation is most pronounced in RP candidates, in whom the favorable selection bias resulted in best OS. Conversely, the magnitude of the survival benefit is less pronounced in EBRT patients. We also observed a striking difference in OS versus predicted LE in non-Hispanic blacks, relative to the three other racial/ethnic groups, regardless of treatment type. In both RP and EBRT groups, Non-Hispanic blacks did not exhibit better OS than predicted LE, like it was displayed in the three other racial/ethnic groups. Instead, non-Hispanic Blacks either perfectly followed their respective LE predictions, or their observed survival was inferior to those predictions. Potential conditions underlying the substantially worse survival of non-Hispanic Black patients should be scrutinized with the intent of eradicating this unfavorable survival pattern of non-Hispanic Black localized PCa patients treated with RP or EBRT, and possibly of non-Hispanic Blacks in general [20, 21]. Future health politics and health care providers need to consider the survival disadvantage of non-Hispanic Blacks, which seems to be also in effect in metastatic PCa also within other tumor entities, such as in renal cell carcinoma [22, 23]. Some investigators suggested that this race/ethnicity-related survival difference would originate from a variety of underlying health issues which may not only derive from potential inherent lifestyle issues, but may also be a part of environmental and occupational disadvantages which need to be addressed by future health politics [24]. Therefore, further research should also elucidate this survival discrepancy beyond the currently investigated tumor entities, and even more so in other countries which also rely on life tables for predicting LE. This is especially relevant in countries like Canada, Switzerland, France, or South Africa, where life tables do not incorporate the factor of race/ethnicity, despite their known heterogeneous population [25–28]. Finally, these findings are very far reaching and are not exclusively applicable to urological practice or to urologic oncology, and should therefore be addressed and investigated in primary and secondary prevention settings.

Despite its novelty, our study has limitations. The first limitation is the nature of the study population, which was diagnosed and treated between 2004 and 2006. The selection of these individuals was dictated by the need of complete 10-year follow-up. In consequence, more contemporary data that had less maturity could not be included. However, it is possible that contemporary non-Hispanic Black patients will no longer exhibit the observed OS disadvantage [29]. Indeed, when considering the analyses by Dess et al. [30] who performed adjustment for nonbiological differences such as access to care and standardized treatment within the Veteran Affairs health system and National Cancer Institute datasets, no discrepancy with regard to prostate cancer–specific mortality between non-Hispanic Black and non-Hispanic White patients remained. These findings were also supported by recent analyses by Stern et al. in the Canadian Health Care system, where universal access to health care is available [31]. Nevertheless, also in the analyses by Dess et al., a disparity in rates of other-cause mortality remained. In this regard, SEER does not provide information on comorbidities, nor access to care. In consequence, we could not perform a more detailed analysis to examine the underlying comorbidity profiles or potentially relevant disparities in health care access according to each racial/ethnic group. Furthermore, the aim of our analyses was to compare predicted LE with OS within each racial/ethnic group. Therefore, no direct statistical comparisons between races/ethnicities were performed. As a result, presented OS survival rates between races/ethnicities should be interpreted with care. Second, although non-Hispanic Whites are well represented in the SEER database, the representation of non-Hispanic Blacks, Hispanic/Latinos, and Asians is suboptimal. Oversampling of these patients should be encouraged in the future, to allow better generalizability of observed findings within samples of non-Hispanic Black, Hispanic/Latino, and Asian men. Nonetheless, despite those observations, also the smallest sample size in this cohort, namely Asian men treated with either RP (*n* = 1241) or EBRT (*n* = 1742), was still adequate. Third, we focused on intermediate and high-risk patients, since these two risk groups represent the optimal patient pool for active treatment [11, 12]. Therefore, our analysis did not include patients treated with active surveillance. However, a small proportion of PCa patients die of their disease, even among high-risk patients [32]. In this regard, it may be argued that non-Hispanic Black men may have exhibited the most unfavorable comorbidity profile, especially in the light of previous data by Dess et al. and Bandini et al., displaying marginal differences in cancer-specific mortality between non-Hispanic White and non-Hispanic Black patients [30, 33]. Finally, although the SEER database samples roughly one-third of the USA population and approximates demographic compositions, there is a tendency towards a relatively higher proportion of urban-based patients and foreign-born patients. Additionally, within the SSA life tables, the Social Security area population is comprised of residents of all 50 States and the District of Columbia, but also residents of Puerto Rico, the Virgin Islands, Guam, American Samoa, and the Northern Mariana Islands; furthermore, also federal employees and US citizens and their dependents who are living abroad [4]. Therefore, a certain mismatch between SEER data and SSA life tables needs to be expected. Apart from that, differences in treatment rates and prostate cancer characteristics, but also potential differences in LE within different SEER registries needs to be considered when interpreting our findings, bearing in mind a potential numerator-denominator bias [34].

## Conclusions

When comparing SEER-derived observed overall survival with SSA life table–derived predicted life expectancy, we recorded a survival disadvantage in non-Hispanic Black RP and EBRT patients, which was not the case in the three other races/ethnicities (non-Hispanic Whites, Hispanic/Latinos, and Asians). This discrepancy should ideally be confirmed within different registries, countries, and tumor entities. Furthermore, the source of these discrepant survival outcomes should be investigated and addressed by health care politics.

## Data Availability

R software environment for statistical computing and graphics (version 3.4.0 for MAC OS X; http://www.r-project.org/) was used for all statistical analyses. Used codes for analyses can be provided. The data that support the findings of this study are available from the Surveillance, Epidemiology and End results database (SEER), but restrictions apply to the availability of these data, which were used under license for the current study, and so are not publicly available. Data are however available from the authors upon reasonable request and with permission of SEER.
